# Ethyl 2-(pyridine-4-carboxamido)-4,5,6,7-tetra­hydro-1-benzothio­phene-3-carboxyl­ate

**DOI:** 10.1107/S1600536812025251

**Published:** 2012-06-13

**Authors:** Asma Mukhtar, M. Nawaz Tahir, Misbahul Ain Khan, Abdul Qayyum Ather, Naveed Sajid

**Affiliations:** a Institute of Chemistry, University of the Punjab, Lahore, Pakistan; b University of Sargodha, Department of Physics, Sargodha, Pakistan; c Department of Chemistry, Islamia University, Bahawalpur, Pakistan; d Applied Chemistry Research Center, PCSIR Laboratories Complex, Lahore 54600, Pakistan

## Abstract

In the title compound, C_17_H_18_N_2_O_3_S, the dihedral angles between the thio­phene ring and the ethyl ester group and the pyridine-4-carboxamide unit are 7.1 (2) and 9.47 (11)°, respectively. An intra­molecular N—H⋯O hydrogen bond generates an *S*(6) ring. In the crystal, inversion dimers linked by pairs of C—H⋯O hydrogen bonds between the tetra­hydro-1-benzothio­phene and the pyridine-4-carboxamide residues generate *R*_2_^2^(16) loops. There exists positional disorder in three methelene groups of the cyclo­hexane ring and the terminal C atom of the ethyl ester side chain in a 0.691 (14):0.309 (14) occupancy ratio.

## Related literature

For related structures, see: Mukhtar *et al.* (2010*a* ▶,*b* ▶).
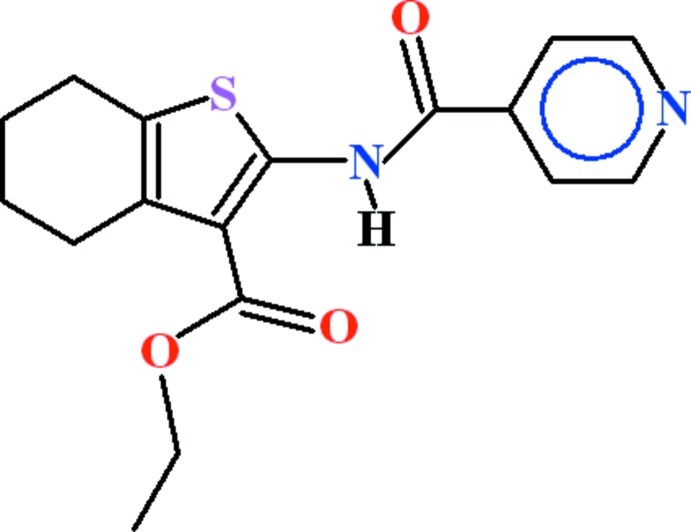


## Experimental

### 

#### Crystal data

C_17_H_18_N_2_O_3_S
*M* *_r_* = 330.39
Triclinic, 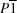

*a* = 8.5604 (6) Å
*b* = 9.3481 (7) Å
*c* = 11.7443 (10) Å
α = 105.121 (3)°
β = 99.748 (2)°
γ = 110.806 (3)°
*V* = 811.59 (11) Å^3^
*Z* = 2
Mo *K*α radiation
μ = 0.22 mm^−1^
*T* = 296 K
0.24 × 0.18 × 0.15 mm

#### Data collection

Bruker Kappa APEXII CCD diffractometer
Absorption correction: multi-scan (*SADABS*; Bruker, 2005 ▶) *T*_min_ = 0.953, *T*_max_ = 0.958
12176 measured reflections
2914 independent reflections
1842 reflections with *I* > 2σ(*I*)
*R*_int_ = 0.039

#### Refinement

*R*[*F* ^2^ > 2σ(*F* ^2^)] = 0.045
*wR*(*F* ^2^) = 0.116
*S* = 1.00
2914 reflections
243 parameters
10 restraints
H-atom parameters constrained
Δρ_max_ = 0.15 e Å^−3^
Δρ_min_ = −0.18 e Å^−3^

### 

Data collection: *APEX2* (Bruker, 2009 ▶); cell refinement: *SAINT* (Bruker, 2009 ▶); data reduction: *SAINT*; program(s) used to solve structure: *SHELXS97* (Sheldrick, 2008 ▶); program(s) used to refine structure: *SHELXL97* (Sheldrick, 2008 ▶); molecular graphics: *ORTEP-3 for Windows* (Farrugia, 1997 ▶) and *PLATON* (Spek, 2009 ▶); software used to prepare material for publication: *WinGX* (Farrugia, 1999 ▶) and *PLATON*.

## Supplementary Material

Crystal structure: contains datablock(s) global, I. DOI: 10.1107/S1600536812025251/hb6838sup1.cif

Structure factors: contains datablock(s) I. DOI: 10.1107/S1600536812025251/hb6838Isup2.hkl

Supplementary material file. DOI: 10.1107/S1600536812025251/hb6838Isup3.cml

Additional supplementary materials:  crystallographic information; 3D view; checkCIF report

## Figures and Tables

**Table 1:** Hydrogen-bond geometry (Å, °)

*D*—H⋯*A*	*D*—H	H⋯*A*	*D*⋯*A*	*D*—H⋯*A*
N1—H1⋯O2	0.86	1.99	2.650 (3)	132
C7*A*—H7*A*⋯O3^i^	0.97	2.56	3.323 (12)	136

Symmetry code: (i) 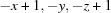.

**Fig. 1.**
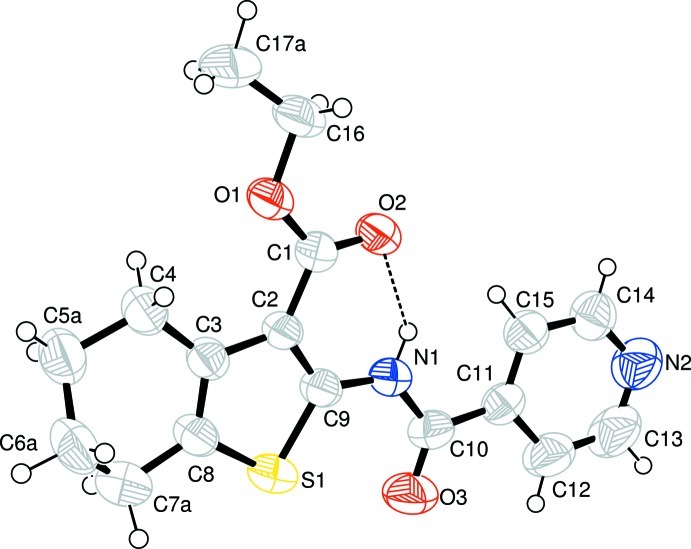
View of the title compound with displacement ellipsoids drawn at the 50% probability level. The dotted line show intramolecular H-bonding.

**Fig. 2.**
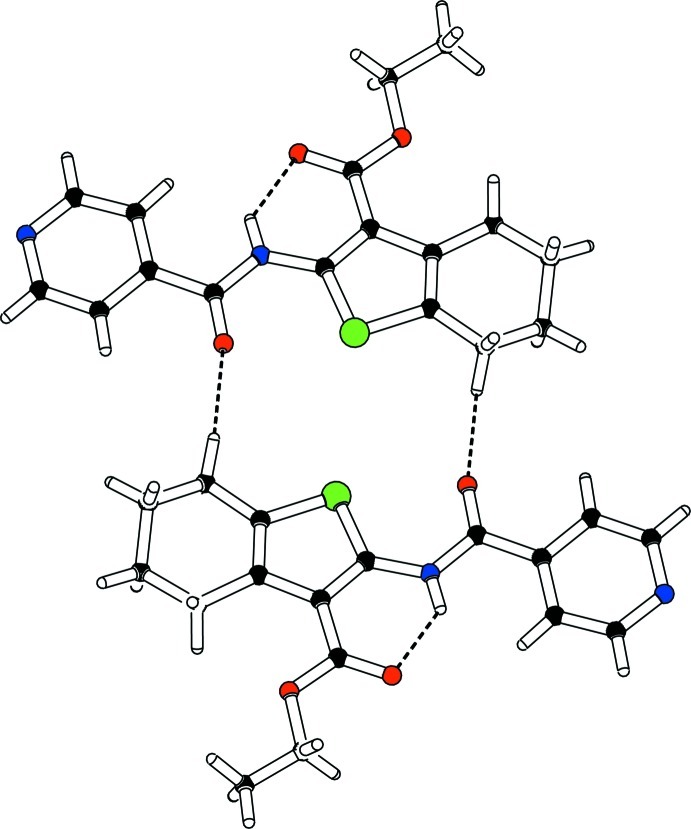
The partial packing, which shows that molecules form dimers with R22(16) ring.

## supplementary crystallographic information

### Comment

We reported the crystal structures of ethyl 2-benzamido-4,5,6,7-tetrahydro-1- benzothiophene-3-carboxylate (Mukhtar *et al.*, 2010*a*) and diethyl 5-acetamido-3-methylthiophene-2,4-dicarboxylate (Mukhtar *et al.*, 2010*b*) which are related to the tile compound (I), (Fig. 1).

In (I), the thiophene ring A (S1/C8/C3/C2/C9), ethyl ester group B (O1/C1/O2/C16/C17A) and pyridine-4-carboxamide moiety C (C10—C15/N1/N2/O3) are planar with r. m. s. deviation of 0.0010, 0.0906 and 0.0520 Å, respectively. The dihedral angle between A/B, A/C and B/C is 7.07 (21), 9.47 (11) and 3.30 (20)°, respectively. In the title compound an S(6) ring motif is formed due to intramolecular H-bonding of N—H···O type (Table 1, Fig. 1). The molecules are linked in the form of dimers with *R*_2_^2^(16) ring motif due to C—H···O type of H-bonding (Table 1, Fig. 2). Three methelene groups of cyclohexane ring and terminal C-atom of ethyl ester are disordered over two set of sites with occupancy ratio of 0.691 (14):0.309 (14).

### Experimental

A mixture of (0.4 g, 3 mmol) of pyridine-4-carboxylic acid and 0.5 ml of thionyl chloride was heated for 5 minutes. Ethyl 2-pyridyl-4-amido-4,5,6,7- tetrahydro-1-benzothiophene-3-carboxylate (0.7 g, 3 mmol) was dissolved in 30 ml chloroform separately and then added to the former mixture. The whole reaction mixture was refluxed for 45 minutes. The solvent was removed and residue was recrystallized in acetone to give colorless prisms of (I). M.p.: 431 K, yield: 0.92 g, 80%.

### Refinement

In the cyclohexane ring three methelene groups and terminal C-atom of ethyl ester are disordered over two set of sites with occupancy ratio of 0.691 (14):0.309 (14).

The H-atoms were positioned geometrically (N—H = 0.86, C–H = 0.93–0.97 Å) and refined as riding with *U*_iso_(H) = *xU*_eq_(C, N), where *x* = 1.5 for methyl and *x* = 1.2 for other H-atoms.

### Crystal data

**Table:**

C_17_H_18_N_2_O_3_S	*Z* = 2
*M**_r_* = 330.39	*F*(000) = 384
Triclinic, *P*1	*D*_x_ = 1.352 Mg m^−^^3^
Hall symbol: -P 1	Mo *K*α radiation, λ = 0.71073 Å
*a* = 8.5604 (6) Å	Cell parameters from 1842 reflections
*b* = 9.3481 (7) Å	θ = 2.5–25.3°
*c* = 11.7443 (10) Å	µ = 0.22 mm^−^^1^
α = 105.121 (3)°	*T* = 296 K
β = 99.748 (2)°	Prism, colorless
γ = 110.806 (3)°	0.24 × 0.18 × 0.15 mm
*V* = 811.59 (11) Å^3^	

### Data collection

**Table:**

Bruker Kappa APEXII CCD diffractometer	2914 independent reflections
Radiation source: fine-focus sealed tube	1842 reflections with *I* > 2σ(*I*)
Graphite monochromator	*R*_int_ = 0.039
Detector resolution: 8.10 pixels mm^-1^	θ_max_ = 25.3°, θ_min_ = 2.5°
ω scans	*h* = −10→8
Absorption correction: multi-scan (*SADABS*; Bruker, 2005)	*k* = −11→11
*T*_min_ = 0.953, *T*_max_ = 0.958	*l* = −14→14
12176 measured reflections	

### Refinement

**Table:**

Refinement on *F*^2^	Primary atom site location: structure-invariant direct methods
Least-squares matrix: full	Secondary atom site location: difference Fourier map
*R*[*F*^2^ > 2σ(*F*^2^)] = 0.045	Hydrogen site location: inferred from neighbouring sites
*wR*(*F*^2^) = 0.116	H-atom parameters constrained
*S* = 1.00	*w* = 1/[σ^2^(*F*_o_^2^) + (0.049*P*)^2^ + 0.1304*P*] where *P* = (*F*_o_^2^ + 2*F*_c_^2^)/3
2914 reflections	(Δ/σ)_max_ < 0.001
243 parameters	Δρ_max_ = 0.15 e Å^−^^3^
10 restraints	Δρ_min_ = −0.18 e Å^−^^3^

### Special details

**Table:**

Geometry. Bond distances, angles *etc*. have been calculated using the rounded fractional coordinates. All su's are estimated from the variances of the (full) variance-covariance matrix. The cell e.s.d.'s are taken into account in the estimation of distances, angles and torsion angles
Refinement. Refinement of *F*^2^ against ALL reflections. The weighted *R*-factor *wR* and goodness of fit *S* are based on *F*^2^, conventional *R*-factors *R* are based on *F*, with *F* set to zero for negative *F*^2^. The threshold expression of *F*^2^ > σ(*F*^2^) is used only for calculating *R*-factors(gt) *etc*. and is not relevant to the choice of reflections for refinement. *R*-factors based on *F*^2^ are statistically about twice as large as those based on *F*, and *R*- factors based on ALL data will be even larger.

### Fractional atomic coordinates and isotropic or equivalent isotropic displacement parameters (Å^2^)

**Table:**

	*x*	*y*	*z*	*U*_iso_*/*U*_eq_	Occ. (<1)
S1	0.57947 (9)	0.19759 (8)	0.46283 (7)	0.0767 (3)	
O1	0.6976 (2)	0.68634 (18)	0.35270 (15)	0.0742 (6)	
O2	0.8949 (2)	0.73080 (19)	0.52291 (15)	0.0730 (6)	
O3	0.8381 (3)	0.2881 (3)	0.68270 (19)	0.1003 (8)	
N1	0.8635 (2)	0.4829 (2)	0.60071 (17)	0.0615 (7)	
N2	1.3610 (3)	0.7692 (4)	0.9953 (2)	0.0968 (11)	
C1	0.7620 (3)	0.6392 (3)	0.4390 (2)	0.0580 (8)	
C2	0.6600 (3)	0.4677 (3)	0.42082 (19)	0.0517 (8)	
C3	0.5015 (3)	0.3510 (3)	0.3252 (2)	0.0568 (8)	
C4	0.4040 (3)	0.3808 (3)	0.2210 (2)	0.0684 (9)	
C5A	0.2613 (14)	0.2210 (11)	0.1232 (9)	0.084 (3)	0.691 (14)
C6A	0.1643 (9)	0.1068 (9)	0.1818 (7)	0.090 (2)	0.691 (14)
C7A	0.2885 (14)	0.0544 (12)	0.2537 (10)	0.093 (5)	0.691 (14)
C8	0.4453 (3)	0.2032 (3)	0.3374 (2)	0.0647 (9)	
C9	0.7149 (3)	0.3990 (3)	0.5005 (2)	0.0571 (8)	
C10	0.9163 (3)	0.4265 (3)	0.6877 (2)	0.0681 (10)	
C11	1.0748 (3)	0.5483 (3)	0.7916 (2)	0.0624 (9)	
C12	1.1234 (4)	0.5047 (4)	0.8909 (3)	0.0915 (12)	
C13	1.2663 (5)	0.6197 (5)	0.9893 (3)	0.1068 (16)	
C14	1.3131 (4)	0.8068 (4)	0.8988 (3)	0.0810 (11)	
C15	1.1737 (3)	0.7024 (3)	0.7966 (2)	0.0690 (10)	
C16	0.7948 (4)	0.8546 (3)	0.3616 (3)	0.0935 (11)	
C17A	0.7281 (10)	0.8628 (10)	0.2439 (6)	0.113 (3)	0.691 (14)
C5B	0.221 (2)	0.242 (2)	0.159 (3)	0.083 (7)	0.310 (14)
C6B	0.225 (2)	0.0756 (16)	0.1323 (18)	0.091 (6)	0.310 (14)
C7B	0.275 (3)	0.054 (2)	0.2566 (18)	0.074 (8)	0.310 (14)
C17B	0.687 (2)	0.894 (2)	0.2657 (6)	0.103 (5)	0.310 (14)
H4A	0.48597	0.43620	0.18225	0.0821*	
H4B	0.35058	0.45180	0.25392	0.0821*	
H1	0.92888	0.58106	0.60821	0.0738*	
H7A	0.23001	−0.00598	0.30131	0.1119*	0.691 (14)
H7B	0.32310	−0.01579	0.19682	0.1119*	0.691 (14)
H12	1.06183	0.40089	0.89190	0.1100*	
H13	1.29763	0.58902	1.05578	0.1283*	
H14	1.37777	0.91097	0.89972	0.0972*	
H15	1.14681	0.73660	0.73125	0.0828*	
H16A	0.77654	0.92982	0.42663	0.1120*	
H16B	0.91878	0.88177	0.37895	0.1120*	
H17A	0.60304	0.82038	0.22283	0.1699*	0.691 (14)
H17B	0.76116	0.79910	0.18235	0.1699*	0.691 (14)
H17C	0.77538	0.97428	0.24783	0.1699*	0.691 (14)
H5A	0.17977	0.24607	0.07281	0.1004*	0.691 (14)
H5B	0.31459	0.16809	0.06985	0.1004*	0.691 (14)
H6A	0.06979	0.01117	0.11859	0.1079*	0.691 (14)
H6B	0.11397	0.16052	0.23733	0.1079*	0.691 (14)
H5C	0.14711	0.25201	0.21193	0.0997*	0.310 (14)
H5D	0.17039	0.25150	0.08217	0.0997*	0.310 (14)
H6C	0.31023	0.06938	0.08848	0.1103*	0.310 (14)
H6D	0.11147	−0.00965	0.08180	0.1103*	0.310 (14)
H7C	0.29232	−0.04536	0.24441	0.0886*	0.310 (14)
H7D	0.18307	0.04701	0.29589	0.0886*	0.310 (14)
H17D	0.56995	0.86379	0.27276	0.1534*	0.310 (14)
H17E	0.68253	0.83476	0.18437	0.1534*	0.310 (14)
H17F	0.73952	1.00931	0.28019	0.1534*	0.310 (14)

### Atomic displacement parameters (Å^2^)

**Table:**

	*U*^11^	*U*^22^	*U*^33^	*U*^12^	*U*^13^	*U*^23^
S1	0.0865 (5)	0.0500 (4)	0.0840 (5)	0.0152 (3)	0.0208 (4)	0.0297 (3)
O1	0.0827 (12)	0.0456 (10)	0.0725 (11)	0.0125 (9)	−0.0016 (9)	0.0224 (8)
O2	0.0732 (11)	0.0510 (10)	0.0704 (11)	0.0079 (9)	−0.0004 (10)	0.0225 (9)
O3	0.1113 (16)	0.0709 (13)	0.1059 (15)	0.0190 (12)	0.0106 (12)	0.0512 (12)
N1	0.0666 (13)	0.0511 (12)	0.0605 (12)	0.0171 (10)	0.0133 (10)	0.0238 (10)
N2	0.0930 (19)	0.110 (2)	0.0823 (17)	0.0380 (17)	0.0115 (14)	0.0410 (16)
C1	0.0664 (16)	0.0493 (14)	0.0562 (14)	0.0225 (13)	0.0151 (13)	0.0193 (12)
C2	0.0573 (14)	0.0414 (13)	0.0528 (13)	0.0167 (11)	0.0177 (11)	0.0152 (10)
C3	0.0581 (14)	0.0496 (14)	0.0562 (14)	0.0177 (12)	0.0194 (12)	0.0133 (11)
C4	0.0658 (16)	0.0581 (15)	0.0681 (16)	0.0203 (13)	0.0112 (13)	0.0147 (12)
C5A	0.074 (4)	0.073 (5)	0.076 (6)	0.019 (3)	0.003 (3)	0.012 (3)
C6A	0.067 (4)	0.072 (4)	0.085 (4)	0.000 (3)	0.003 (3)	0.008 (3)
C7A	0.090 (9)	0.074 (8)	0.098 (9)	0.010 (6)	0.028 (7)	0.037 (6)
C8	0.0640 (16)	0.0470 (15)	0.0688 (16)	0.0117 (13)	0.0176 (13)	0.0156 (12)
C9	0.0635 (15)	0.0458 (13)	0.0590 (14)	0.0177 (12)	0.0229 (13)	0.0178 (12)
C10	0.0803 (18)	0.0627 (17)	0.0696 (17)	0.0299 (15)	0.0245 (14)	0.0343 (14)
C11	0.0681 (16)	0.0712 (18)	0.0609 (15)	0.0365 (14)	0.0223 (13)	0.0306 (13)
C12	0.096 (2)	0.098 (2)	0.086 (2)	0.0341 (19)	0.0211 (19)	0.0535 (19)
C13	0.108 (3)	0.134 (3)	0.083 (2)	0.048 (2)	0.010 (2)	0.060 (2)
C14	0.0760 (19)	0.084 (2)	0.0749 (19)	0.0317 (16)	0.0091 (16)	0.0260 (16)
C15	0.0719 (17)	0.0702 (17)	0.0687 (17)	0.0327 (15)	0.0147 (14)	0.0295 (14)
C16	0.108 (2)	0.0462 (16)	0.097 (2)	0.0126 (15)	−0.0073 (17)	0.0308 (15)
C17A	0.155 (6)	0.062 (5)	0.118 (5)	0.040 (4)	0.010 (4)	0.050 (4)
C5B	0.071 (11)	0.066 (9)	0.081 (14)	0.023 (8)	−0.005 (7)	0.001 (8)
C6B	0.073 (9)	0.074 (9)	0.098 (12)	0.025 (6)	0.004 (7)	0.006 (7)
C7B	0.060 (13)	0.027 (10)	0.083 (18)	−0.002 (9)	0.000 (11)	−0.019 (10)
C17B	0.151 (12)	0.052 (7)	0.076 (8)	0.055 (8)	−0.032 (7)	0.002 (5)

### Geometric parameters (Å, º)

**Table:**

S1—C8	1.733 (3)	C16—C17A	1.436 (8)
S1—C9	1.711 (3)	C16—C17B	1.537 (14)
O1—C1	1.320 (3)	C4—H4A	0.9700
O1—C16	1.460 (3)	C4—H4B	0.9700
O2—C1	1.219 (3)	C5A—H5A	0.9700
O3—C10	1.207 (4)	C5A—H5B	0.9700
N1—C9	1.386 (3)	C5B—H5D	0.9700
N1—C10	1.349 (3)	C5B—H5C	0.9700
N2—C13	1.319 (6)	C6A—H6B	0.9700
N2—C14	1.315 (4)	C6A—H6A	0.9700
N1—H1	0.8600	C6B—H6D	0.9700
C1—C2	1.464 (4)	C6B—H6C	0.9700
C2—C3	1.444 (3)	C7A—H7B	0.9700
C2—C9	1.374 (4)	C7A—H7A	0.9700
C3—C4	1.506 (3)	C7B—H7D	0.9700
C3—C8	1.347 (4)	C7B—H7C	0.9700
C4—C5B	1.53 (2)	C12—H12	0.9300
C4—C5A	1.542 (11)	C13—H13	0.9300
C5A—C6A	1.500 (13)	C14—H14	0.9300
C5B—C6B	1.52 (3)	C15—H15	0.9300
C6A—C7A	1.538 (14)	C16—H16A	0.9700
C6B—C7B	1.54 (3)	C16—H16B	0.9700
C7A—C8	1.489 (12)	C17A—H17C	0.9600
C7B—C8	1.54 (2)	C17A—H17A	0.9600
C10—C11	1.498 (3)	C17A—H17B	0.9600
C11—C12	1.380 (4)	C17B—H17D	0.9600
C11—C15	1.367 (4)	C17B—H17E	0.9600
C12—C13	1.388 (5)	C17B—H17F	0.9600
C14—C15	1.376 (4)		
			
C8—S1—C9	90.88 (13)	H5A—C5A—H5B	108.00
C1—O1—C16	116.6 (2)	C4—C5B—H5C	109.00
C9—N1—C10	126.8 (2)	C4—C5B—H5D	109.00
C13—N2—C14	115.6 (3)	C6B—C5B—H5C	109.00
C10—N1—H1	117.00	C6B—C5B—H5D	109.00
C9—N1—H1	117.00	H5C—C5B—H5D	108.00
O1—C1—C2	113.0 (2)	H6A—C6A—H6B	108.00
O2—C1—C2	124.7 (2)	C5A—C6A—H6A	110.00
O1—C1—O2	122.3 (2)	C5A—C6A—H6B	110.00
C3—C2—C9	111.7 (2)	C7A—C6A—H6A	110.00
C1—C2—C3	128.5 (2)	C7A—C6A—H6B	110.00
C1—C2—C9	119.8 (2)	C7B—C6B—H6C	110.00
C4—C3—C8	121.2 (2)	C7B—C6B—H6D	110.00
C2—C3—C8	111.9 (2)	C5B—C6B—H6D	110.00
C2—C3—C4	126.9 (2)	C5B—C6B—H6C	110.00
C3—C4—C5B	110.8 (10)	H6C—C6B—H6D	108.00
C3—C4—C5A	112.2 (4)	C6A—C7A—H7A	110.00
C4—C5A—C6A	111.4 (7)	C6A—C7A—H7B	110.00
C4—C5B—C6B	111.8 (14)	H7A—C7A—H7B	108.00
C5A—C6A—C7A	110.2 (8)	C8—C7A—H7B	110.00
C5B—C6B—C7B	107.5 (18)	C8—C7A—H7A	110.00
C6A—C7A—C8	108.7 (8)	C6B—C7B—H7C	110.00
C6B—C7B—C8	107.5 (13)	C8—C7B—H7D	110.00
C3—C8—C7B	125.6 (8)	C8—C7B—H7C	110.00
C3—C8—C7A	126.1 (5)	C6B—C7B—H7D	110.00
S1—C8—C3	112.96 (19)	H7C—C7B—H7D	109.00
S1—C8—C7A	121.0 (5)	C11—C12—H12	121.00
S1—C8—C7B	121.3 (8)	C13—C12—H12	121.00
S1—C9—N1	123.36 (19)	C12—C13—H13	118.00
S1—C9—C2	112.64 (19)	N2—C13—H13	118.00
N1—C9—C2	124.0 (2)	C15—C14—H14	118.00
N1—C10—C11	115.4 (2)	N2—C14—H14	118.00
O3—C10—C11	122.7 (2)	C11—C15—H15	120.00
O3—C10—N1	121.8 (2)	C14—C15—H15	120.00
C10—C11—C15	124.5 (2)	O1—C16—H16A	111.00
C10—C11—C12	118.4 (3)	O1—C16—H16B	111.00
C12—C11—C15	117.1 (2)	C17A—C16—H16A	111.00
C11—C12—C13	118.4 (3)	C17A—C16—H16B	111.00
N2—C13—C12	124.8 (3)	H16A—C16—H16B	109.00
N2—C14—C15	124.4 (3)	C17B—C16—H16A	89.00
C11—C15—C14	119.7 (3)	C17B—C16—H16B	126.00
O1—C16—C17B	108.9 (7)	C16—C17A—H17A	109.00
O1—C16—C17A	105.7 (4)	C16—C17A—H17B	109.00
C3—C4—H4A	109.00	C16—C17A—H17C	109.00
C3—C4—H4B	109.00	H17A—C17A—H17B	109.00
C5A—C4—H4A	109.00	H17A—C17A—H17C	109.00
C5A—C4—H4B	109.00	H17B—C17A—H17C	110.00
H4A—C4—H4B	108.00	C16—C17B—H17D	110.00
C5B—C4—H4A	128.00	C16—C17B—H17E	110.00
C5B—C4—H4B	89.00	C16—C17B—H17F	110.00
C4—C5A—H5A	109.00	H17D—C17B—H17E	109.00
C4—C5A—H5B	109.00	H17D—C17B—H17F	109.00
C6A—C5A—H5A	109.00	H17E—C17B—H17F	109.00
C6A—C5A—H5B	109.00		
			
C9—S1—C8—C3	0.1 (2)	C3—C2—C9—S1	−0.2 (3)
C9—S1—C8—C7A	178.7 (6)	C3—C2—C9—N1	179.9 (2)
C8—S1—C9—N1	−180.0 (2)	C2—C3—C4—C5A	169.0 (5)
C8—S1—C9—C2	0.1 (2)	C8—C3—C4—C5A	−10.4 (6)
C16—O1—C1—O2	0.6 (4)	C2—C3—C8—S1	−0.2 (3)
C16—O1—C1—C2	−178.1 (2)	C2—C3—C8—C7A	−178.7 (6)
C1—O1—C16—C17A	165.1 (4)	C4—C3—C8—S1	179.3 (2)
C10—N1—C9—S1	−5.8 (4)	C4—C3—C8—C7A	0.8 (7)
C10—N1—C9—C2	174.1 (2)	C3—C4—C5A—C6A	41.5 (9)
C9—N1—C10—O3	3.3 (4)	C4—C5A—C6A—C7A	−63.6 (10)
C9—N1—C10—C11	−175.2 (2)	C5A—C6A—C7A—C8	51.1 (10)
C14—N2—C13—C12	−0.6 (6)	C6A—C7A—C8—S1	160.6 (5)
C13—N2—C14—C15	0.6 (5)	C6A—C7A—C8—C3	−21.0 (10)
O1—C1—C2—C3	−1.7 (4)	O3—C10—C11—C12	−6.1 (4)
O1—C1—C2—C9	177.4 (2)	O3—C10—C11—C15	175.4 (3)
O2—C1—C2—C3	179.6 (3)	N1—C10—C11—C12	172.3 (3)
O2—C1—C2—C9	−1.3 (4)	N1—C10—C11—C15	−6.2 (4)
C1—C2—C3—C4	−0.1 (4)	C10—C11—C12—C13	−177.6 (3)
C1—C2—C3—C8	179.4 (3)	C15—C11—C12—C13	1.0 (5)
C9—C2—C3—C4	−179.3 (2)	C10—C11—C15—C14	177.5 (3)
C9—C2—C3—C8	0.2 (3)	C12—C11—C15—C14	−1.0 (4)
C1—C2—C9—S1	−179.41 (19)	C11—C12—C13—N2	−0.3 (6)
C1—C2—C9—N1	0.6 (4)	N2—C14—C15—C11	0.2 (5)

### Hydrogen-bond geometry (Å, º)

**Table:**

*D*—H···*A*	*D*—H	H···*A*	*D*···*A*	*D*—H···*A*
N1—H1···O2	0.86	1.99	2.650 (3)	132
C7*A*—H7*A*···O3^i^	0.97	2.56	3.323 (12)	136

Symmetry code: (i) −*x*+1, −*y*, −*z*+1.
